# MRI-Based Prediction of Vestibular Schwannoma: Systematic Review

**DOI:** 10.3390/cancers18020289

**Published:** 2026-01-17

**Authors:** Cheng Yang, Daniel Alvarado, Pawan Kishore Ravindran, Max E. Keizer, Koos Hovinga, Martinus P. G. Broen, Henricus P. M. Kunst, Yasin Temel

**Affiliations:** 1 Department of Neurosurgery, Maastricht University Medical Center, 6202 AZ Maastricht, The Netherlands; daniel.alvarado@mumc.nl (D.A.); max.keizer@mumc.nl (M.E.K.);; 2 Dutch Academic Alliance Skull Base Pathology, Maastricht University Medical Center, 6202 AZ Maastricht, The Netherlands; 3 Department of Neurology, Maastricht University Medical Center, 6202 AZ Maastricht, The Netherlands; 4 Department of Otorhinolaryngology, Maastricht University Medical Center, 6202 AZ Maastricht, The Netherlands; 5 Department of Otorhinolaryngology, Radboud University Medical Center, 6525 GA Nijmegen, The Netherlands; 6 Dutch Academic Alliance Skull Base Pathology, Radboud University Medical Center, 6525 GA Nijmegen, The Netherlands; 7 Istanbul Atlas University, 34406 Istanbul, Turkey

**Keywords:** systematic review, vestibular schwannoma, growth, MRI, skull base tumor, predictor

## Abstract

**Background**: The vestibular schwannoma (VS) is the most common cerebellopontine angle tumor in adults, exhibiting a highly variable natural history, from stability to rapid growth. Accurate, the non-invasive prediction of tumor behavior is essential to guide personalized management and avoid overtreatment or delayed intervention. **Objective**: To systematically review and synthesize the evidence on MRI-based biomarkers for predicting VS growth and treatment responses. **Methods**: We conducted a PRISMA-compliant search of PubMed, EMBASE, and Cochrane databases for studies published between 1 January 2000 and 1 January 2025, addressing MRI predictors of VS growth. Cohort studies evaluating texture features, signal intensity ratios, perfusion parameters, and apparent diffusion coefficient (ADC) metrics were included. Study quality was assessed using the NOS (Newcastle–Ottawa Scale) score, GRADE (Grading of Recommendations, Assessment, Development and Evaluation), and ROBIS (Risk of Bias in Systematic reviews) tool. Data on diagnostic performance, including the area under the receiver operating characteristic (ROC) curve (AUC), sensitivity, specificity, and *p* value, were extracted and descriptively analyzed. **Results**: Ten cohort studies (five retrospective, five prospective, total *n* = 525 patients) met the inclusion criteria. Texture analysis metrics, such as kurtosis and gray-level co-occurrence matrix (GLCM) features, yielded AUCs of 0.65–0.99 for predicting volumetric or linear growth thresholds. Signal intensity ratios on gadolinium-enhanced T1-weighted images for tumor/temporalis muscle achieved a 100% sensitivity and 93.75% specificity. Perfusion MRI parameters (K^trans^, v_e_, ASL, and DSC derived blood-flow metrics) differentiated growing from stable tumors with AUCs up to 0.85. ADC changes post-gamma knife surgery predicted a favorable response, though the baseline ADC had limited value for natural growth prediction. The heterogeneity in growth definitions, MRI protocols, and retrospective designs remains a key limitation. **Conclusions**: MRI-based biomarkers may provide exploratory signals associated with VS growth and treatment responses. However, substantial heterogeneity in growth definitions and MRI protocols, small single-center cohorts, and the absence of external validation currently limit clinical implementation.

## Simple Summary

A vestibular schwannoma is a benign tumor that can behave very differently across patients. While some tumors remain stable for many years, others may grow and require treatment. At present, clinicians rely on repeated MRI scans to monitor these tumors, but it is still difficult to determine early on which tumors are likely to grow. The objective of our research was to review and summarize existing studies on MRI-based biomarkers that might improve this prediction. These biomarkers include texture features, signal intensity ratios, perfusion parameters, and apparent diffusion coefficients. We found that these imaging features show promise for identifying tumors that are more likely to grow or to respond well to therapy. However, the available evidence is heterogeneous, largely based on small single-center cohorts, and lacks external validation. Therefore, these MRI-based markers should currently be regarded as exploratory tools rather than predictors ready for routine clinical use.

## 1. Introduction

A vestibular schwannoma (VS), also known as an acoustic neuroma, is a benign, typically slow-growing tumor that arises from Schwann cells of the vestibular portion of the eighth cranial nerve. It accounts for approximately 6–8% of all intracranial tumors and is the most common lesion in the cerebellopontine angle in adults [1]. With the widespread use of magnetic resonance imaging (MRI), the incidental detection rate of VSs has increased significantly over the past two decades [2]. Many patients are now diagnosed at an earlier stage, often before the onset of significant symptoms.

Despite early diagnosis, the clinical decision-making for VSs remains complex, primarily due to the highly variable natural history of these tumors [3]. Some VSs exhibit indolent behavior, remaining radiographically stable for years or even spontaneously regressing. In contrast, others demonstrate sustained growth and the progressive involvement of surrounding structures, leading to debilitating neurological sequelae, such as sensorineural hearing loss, facial nerve palsy, imbalance, and even brainstem compression in advanced cases [4]. The accurate prediction of tumor growth is therefore critical for guiding individualized treatment strategies, as it allows clinicians to tailor monitoring intervals, offer timely interventions, and avoid overtreatment in cases likely to remain stable. However, the prediction of tumor growth remains a major clinical challenge. Traditional risk factors, such as the tumor size, age, and symptoms, show a limited and inconsistent prognostic performance across cohorts [5]. Moreover, these factors often reflect baseline tumor burdens or patient characteristics rather than the underlying biological drivers of growth. Accordingly, more dependable predictors are needed to enable a clinically meaningful risk stratification.

In this context, MRI has emerged not only as a diagnostic tool but also as a potential source of quantitative imaging biomarkers. It provides a non-invasive window into the tumor microstructure, vascularity, and tissue heterogeneity, potentially capturing biological behavior not reflected by clinical variables alone. Alongside ongoing methodological developments, incremental advances in MRI-related techniques have further broadened the scope of measurable imaging features, reinforcing the potential of MRI as a platform for quantitative biomarker exploration [6]. However, the evidence on MRI-based predictors of VS growth remains fragmented and methodologically heterogeneous, with inconsistent growth definitions, variable MRI acquisition protocols, and limited external validation. Therefore, we aim to synthesize MRI-based biomarkers for potentially predicting VS growth and treatment responses, critically appraise methodological limitations, and identify priorities for future prospective multicenter validation.

## 2. Materials and Methods

The protocol for this review can be found in the PROSPERO online database of systematic reviews (ID: CRD420251057619).

### 2.1. Search Strategy

This systematic review adhered to the PRISMA (Preferred Reporting Items for Systematic Reviews and Meta-Analyses) guidelines. A comprehensive literature search was conducted across PubMed, EMBASE, and the Cochrane Library using the terms ((Acoustic Neuroma) OR (vestibular schwannoma)) AND ((growth) OR (prediction)) AND (MRI), covering studies published from 1 January 2000 to 1 January 2025. Retrieved records were imported into EndNote (Version 20) (Clarivate Analytics) for reference management, and duplicate entries were subsequently removed. Two authors (C.Y. and D.A.) independently performed an initial screening based on titles and abstracts. Studies deemed potentially relevant underwent a full-text assessment by the same two reviewers to determine eligibility based on predefined inclusion criteria. A third author (Y.T.) independently verified the selection process, and any disagreements were resolved through group consensus.

### 2.2. Study Selection

Eligibility criteria were defined based on the PICOS framework—Population, Issue of Interest, Comparison, Outcome, and Study Design [7]. The population (P) included individuals diagnosed with VSs. The primary issue (I) addressed was tumor growth. Comparisons (C) were conducted between cases showing stable or shrinking tumors. The outcome (O) of interest focused on the MRI biomarkers’ prediction effect, and only cohort-type studies (S) were considered. Studies primarily based on artificial intelligence or machine learning models were not included, as this review focused on interpretable MRI biomarkers rather than model-based growth prediction frameworks.

Additionally, studies were included if (1) they represented original, peer-reviewed research involving MRI-based predictors with VS patients, (2) they reported data on tumor growth and MRI metrics, and (3) they were published in English. Studies were excluded if they (1) involved patients with neurofibromatosis type II; (2) were review articles, meta-analyses, case reports, editorials, books, information pages, or involved animal or phantom models; or (3) were published in languages other than English.

### 2.3. Data Collection

Two authors (C.Y. and D.A.) independently extracted relevant information from each eligible study. To ensure reliability and consistency, a third author (Y.T.) reviewed all extracted data. The following details were systematically collected: author names, year of publication, sample size, demographic characteristics, follow-up time, tumor growth definition, MRI field strength, and key metrics. We used tumor growth to refer to an increase in tumor size over time, including natural tumor growth during wait and scan times and post-treatment tumor growth following surgery or radiotherapy. Treatment response refers to post-treatment tumor shrinkage or transient tumor enlargement (TTE), as reported by the original studies. Linear and volumetric growth refer to diameter- and volume-based measurement approaches. If specific information was not reported or could not be inferred from the text, the corresponding fields in the extraction sheet were marked with a slash (/) to denote missing or unmentioned data. In cases where multiple publications from the same research group potentially included overlapping patient populations, the version with the most complete dataset was selected to avoid duplication.

### 2.4. Quality Assessment

We assessed heterogeneity descriptively, focusing on differences in MRI field strength and definitions of tumor growth outcomes across studies. The quality in individual studies was evaluated using the Newcastle–Ottawa Scale (NOS) [8], which examines three core aspects: study group selection, group comparability, and outcome assessment. Each study was scored on a nine-point scale based on eight specific criteria. Publications receiving a score of 7 or above were categorized as high-quality, reflecting adequate methodological rigor.

To address sensitivity and minimize potential bias, we excluded studies scoring below 5 on the NOS and carefully avoided including multiple publications that may have reported overlapping patient populations. For the final analysis, only studies of moderate to high methodological quality (NOS ≥ 6) were retained. Moreover, we rated the overall certainty of evidence using the GRADE (Grading of Recommendations, Assessment, Development and Evaluation) approach [9,10]. Certainty was assessed for each MRI biomarker domain. We considered five domains, including risk of bias, inconsistency, indirectness, imprecision, and publication bias, and assigned an overall certainty rating using high, moderate, low, or very low. Finally, we assessed the risk of bias in the review process using the ROBIS (Risk of Bias in Systematic Reviews) tool [11], focusing on eligibility criteria, study identification and selection, data collection and study appraisal, synthesis, and findings. The assessments were independently performed by two authors (C.Y. and D.A.), with any discrepancies resolved by a third reviewer (Y.T.) through discussion and consensus.

### 2.5. Statistical Analysis

A meta-analysis was not performed due to substantial clinical and methodological heterogeneity across studies, including inconsistent growth definitions, variable MRI field strengths, and non-uniform follow-up intervals. Pooling such data could yield misleading summary estimates; therefore, we conducted a structured qualitative synthesis, organizing results by biomarker domain and explicitly summarizing and discussing heterogeneity in growth definitions and MRI field strengths across studies.

Descriptive analyses were conducted using IBM SPSS Statistics version 28 (IBM Corp., Armonk, NY, USA). Continuous variables were summarized using means, standard deviations, and ranges. Key metrics will be presented in tables to provide a clear overview of the study findings.

## 3. Results

### 3.1. Characteristics of Inclusion in the Study

**Figure 1:**
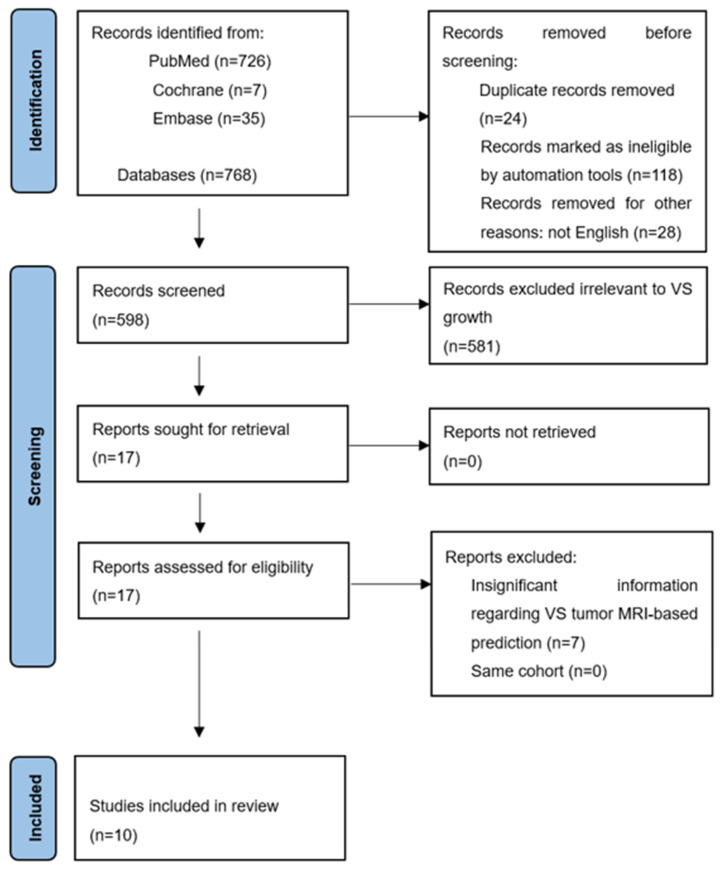
PRISMA 2020 diagram showing the inclusion process.

Our systematic search yielded a total of 768 unique records, with 726 articles retrieved from PubMed, 35 from EMBASE, and 7 from the Cochrane Library. After the initial automatic filtering based on the predefined inclusion and exclusion criteria, 170 records were excluded for reasons such as being non-original articles, duplicate reports, and irrelevant publication types. Subsequently, 598 articles underwent title and abstract screening. At this stage, 581 studies were excluded for lacking relevance to VS growth prediction, either because they did not focus on predictive imaging features, failed to report growth outcomes, or involved type II VSs. This screening process left 17 studies for full-text assessment. After a thorough review of these articles, seven were further excluded because they did not provide sufficient or directly relevant information on tumor growth outcomes in VS patients (see Figure 1 for PRISMA flow diagram).

Ultimately, ten studies met all eligibility criteria and were included in the final qualitative synthesis. Among these, five were retrospective cohort studies and five were prospective cohort studies. All ten studies were single-center investigations; none involved multicenter collaboration. Notably, the reporting of overfitting mitigation methods was inconsistently applied: only one texture study [12] explicitly reported the number of extracted features relative to the sample size, five studies [12,13,14,15,16] employed internal validation, and no study explicitly reported validation in an independent external cohort. Detailed characteristics of the included studies, including the study design, patient population, follow-up duration, and key outcomes, are summarized in Table 1.

This figure shows the flowchart of the literature search, with a total of 768 articles searched from PubMed, Cochrane, and Embase databases. After removing duplicate articles and screening according to inclusion and exclusion criteria, 598 articles were obtained. We read the title and abstract of each paper, excluding 581 irrelevant articles. We read the remaining 17 articles in detail, excluded 7 articles unrelated to tumor growth rate and MRI metrics, and included the remaining 10 articles in this study.

This table summarizes key characteristics of the included studies evaluating vestibular schwannoma growth. The “growth number” refers to the number of tumors classified as growing according to the definition used in each study. The “mean (median) FU/MRI interval *” indicates the follow-up time and the average time between serial MRI scans. Growth measurements were defined by linear or volumetric thresholds, specified individually by study. The MRI field strength indicates the field strength of the imaging used. “/” denotes that the information was not reported. FU = follow-up; IQR = interquartile range; y = years; Mo = months; T = Telsa; and mm^3^ = cubic millimeters.

### 3.2. Study Quality and Risk of Bias

The included studies demonstrated considerable clinical and methodological heterogeneity, particularly with respect to the criteria used to define tumor growth and differences in MRI field strength. These inconsistencies posed challenges for a direct comparison across studies and further supported our decision to forgo a quantitative meta-analysis.

#### 3.2.1. Heterogeneity Evaluation

Across the included studies, growth outcomes were defined using heterogeneous criteria. One study used a linear-only definition [12]. Three studies used volumetric definitions, including one absolute threshold [17] and two relative thresholds [13,14]. One study applied composite criteria [18], and one study used a subject-specific calculator [15]. Three studies did not report an explicit growth definition [19,20,21], and one study reported a TTE [16]. Regarding the MRI field strength, three studies used 3 Telsa (T) scanners [15,18,20], two used 1.5 T scanners [19,21], one reported using both 1.5 T and 3 T systems [17], and four studies did not report field strength [12,13,14,16]. As a result, this heterogeneity limited the direct cross-study comparison of reported performance estimates.

#### 3.2.2. NOS and Bias Evaluation

All ten studies underwent quality appraisal using the NOS score, with results summarized in Supplementary Table S1. Five studies achieved scores of seven or higher and were thus considered high-quality, reflecting robust designs and reporting standards. Five studies received a score of six and thus were considered to be of moderate quality. Importantly, no studies with an NOS score below five were included in this review, in accordance with our exclusion criteria aimed at minimizing methodological bias.

Publication bias was assessed qualitatively, as a formal statistical test was precluded by the synthesis design and limited number of studies. Although our search included gray literature sources, conference abstract archives, and trial registries, all included studies reported at least one statistically significant association for an imaging predictor. This pattern raises concerns about publication bias and suggests that the reported predictive performance may be overestimated. In addition, we did not contact authors to obtain unpublished analyses. The ROBIS assessment indicated some concerns specifically regarding the identification and selection of studies and syntheses and findings (see Table S2).

#### 3.2.3. GRADE Assessment

Using the GRADE approach, the certainty of evidence for texture features and signal intensity metrics was rated as very low, mainly because most included studies were retrospective, single-center, and at risk of overfitting, with substantial heterogeneity in growth definitions and MRI protocols and small sample sizes. In contrast, the certainty for the perfusion parameters and apparent diffusion coefficient (ADC) was rated as low, largely due to methodological and clinical heterogeneity and the predominance of small, single-center cohorts. The domain-level certainty ratings and primary reasons for downgrading are summarized in Table S3.

### 3.3. Key Findings

#### 3.3.1. MRI Texture Features

Five studies investigated the role of MRI texture features in predicting VS treatment outcomes. Speckter et al. [20] identified that the kurtosis of T2-weighted image intensity values, a first-order statistical feature, was the strongest predictor of tumor growth, with a sensitivity of 71% and a specificity of 78%. Three studies [13,14,16] utilized gray-level co-occurrence matrix (GLCM) features to predict significant tumor extension and TTE, reporting an overall AUC ranging from 0.65 to 0.99. Notably, the predictive performance was enhanced for larger tumors (>5 cm^3^ or >6 cm^3^), achieving an accuracy and sensitivity over 80%. Itoyama et al. [12] demonstrated that a high inverse difference moment normalized (Idmn) value was the most effective predictor of rapid VS growth (*p* = 0.003) (see Table 2A).

Table 2A–D outline the imaging technologies, predictive targets, and key metrics used in the included studies. “Technology” refers to the main MRI-based method or image feature applied. “Prediction” denotes the primary clinical outcome being predicted. “Key metrics” include performance indicators such as the AUC, sensitivity, specificity, and relevant thresholds.

#### 3.3.2. MRI Signal Intensity

Three studies examined the correlation between MRI signal characteristics and VS growth behavior. Yamada et al. [17], utilizing gadolinium-enhanced T1-weighted MRI, found that the tumor-to-normal tissue (T/N) ratio, specifically the tumor-to-temporalis muscle (T/Nm) ratio, was significantly higher in growing VSs compared to non-growing tumors, with a sensitivity of 100% and a specificity of 93.75%. Itoyama et al. [12], employing a tumor radiomics analysis, identified that a low “minimum signal value” in contrast-enhanced T1-weighted images, indicative of cystic changes or low enhancement areas, was an independent predictor of rapid tumor growth (*p* = 0.016). Speckter et al. [20] explored radiotherapy responses, demonstrating that the minimum signal value of T2-weighted images was negatively correlated with the final tumor volume reduction (see Table 2B).

#### 3.3.3. Perfusion MRI

Three studies assessed the utility of perfusion MRI in predicting VS growth. Kleijwegt et al. [18] reported that growing VSs, particularly those with a diameter ≥ 9 mm, exhibited distinct hyperintense perfusion characteristics on arterial spin labeling (ASL) and dynamic susceptibility contrast (DSC) MRI, highlighting their vascularization, whereas conventional anatomical MRI only showed a contrast enhancement without a direct correlation to tumor growth. Sammy et al. [15] demonstrated that dynamic contrast-enhanced MRI (DCE-MRI) parameters, including K^trans^ and v_e_, were significantly associated with VS growth, achieving a model AUC of 0.85, effectively distinguishing growing from non-growing tumors. Lewis et al. [21] also confirmed this finding, demonstrating that growing tumors also displayed significantly higher mean tumor K^trans^ values than static tumors using an analysis of variance (ANOVA) (*p* = 0.004) (see Table 2C).

#### 3.3.4. Apparent Diffusion Coefficient

Two studies investigated the potential of ADCs for predicting VS growth. Chuang et al. [19] found that following gamma knife surgery, an increase in ADC values in solid VSs was predictive of a favorable treatment response. Even in cases of transient tumor enlargement, the ADC increase was attributed to radiation effects rather than actual growth. Conversely, cystic VSs exhibited a decrease in ADC values, reflecting a reduction in cystic tumors. Sammy et al. [15] reported that high ADC values were significantly associated with tumor shrinkage (*p* = 0.04) but were not predictive of natural tumor growth (*p* = 0.14) (See Table 2D).

## 4. Discussion

This systematic review evaluated ten studies to synthesize the current evidence on MRI-based predictors of VS growth. The findings underscore the increasing potential of advanced MRI techniques for stratifying tumor behavior and informing individualized management strategies. Across these studies, multiple MRI-based parameters have demonstrated promising predictive value for identifying growing tumors. However, the overall certainty of evidence is limited by the different methodologies used across studies.

We found that the kurtosis of MRI texture features and the GLCM perform well in predicting VS growth, with reported AUC values ranging from 0.65 to 0.99. These imaging features may be associated with the complexity of the internal structure of the tumor and tissue heterogeneity to a certain extent [22]. High GLCM values may capture patterns consistent with irregular tissue structures, cystic changes, microhemorrhages, or necrotic areas of the tumor, which may be associated with faster tumor growth. Tonoyan’s research [23] showed that the kurtosis parameter has been confirmed to be positively correlated with another brain tumor proliferation activity: in gliomas, kurtosis increases with increasing tumor proliferation activity, with a correlation coefficient as high as r = 0.81 (*p* < 0.001). From a biological perspective, increased kurtosis may similarly reflect a higher cell density or proliferation activity in VSs, although the direct histopathological validation remains limited. Identifying such aggressive features non-invasively could have major clinical implications.

Enhanced signal intensity and ratios may provide earlier imaging profiles of more active tumor behavior in VSs. Notably, the T/Nm ratio in growing VSs was significantly higher than that in non-growing VSs, with a sensitivity of 100% and a specificity of 93.75%. This may be because growing VSs may exhibit a higher vascular density and permeability [21], resulting in the increased absorption of gadolinium in the tumor, while the temporalis muscle is not affected by confounding factors such as edema and inflammation compared with brain parenchyma tissues, and its signal intensity is consistent and does not change due to gadolinium enhancement, amplifying the contrast difference in images. This may partly explain why the T/Nm ratio performs well in growing and non-growing tumors. Similarly, a biomarker analysis of VSs found that a lower minimum T1 signal is a predictor of rapid growth. This is consistent with previous studies, suggesting that cystic changes may be associated with more active growth [24]. Meanwhile, a lower minimum signal value on T2-weighted images may be associated with a smaller volume reduction, which is also similar to previous studies that show that cystic tumors have a better response to radiotherapy compared with solid tumors [25]. However, this association requires validation in larger prospective cohorts.

Likewise, perfusion imaging complements the vascular information by reflecting the microvascular environment of the tumor, with growing tumors potentially requiring a greater blood supply to support proliferation. Higher perfusion parameters indicate more active vascularization, which may be associated with the tumor’s growth potential. These perfusion characteristics are consistent with known neuro-oncology biomarkers: for example, K^trans^ are significantly higher in atypical meningiomas than in benign meningiomas, consistent with increased microvascular permeability [26]. In summary, from a biological perspective, both signal enhancement and perfusion may offer indirect information related to tumor vascularity and permeability. Contrast enhancement ratios and perfusion maps provide complementary biomarkers; their combined use for VSs may potentially support the earlier identification of tumors with imaging features associated with faster growth.

ADC values demonstrated context-dependent utility. Following radiosurgery, increased ADC values were associated with favorable treatment responses in solid VSs, likely reflecting reduced cellular density or treatment-induced necrosis. Meanwhile, in cystic VSs, decreases in the ADC were observed in association with spontaneous tumor regression, potentially due to fluid resorption or cyst collapse. However, in the context of untreated tumor growth, ADC values did not show significant predictive value (*p* = 0.14). These findings suggest that the ADC may be more informative for monitoring post-treatment changes rather than serving as a reliable biomarker for predicting intrinsic tumor growth.

Clinically, these metrics could potentially translate into follow-up strategies. The intensity ratio markers with very high sensitivity and specificity may serve as triage tools for intensified follow-up, whereas the biomarkers with a moderate AUC value are better suited for risk-tiering, especially in large-volume tumors. Perfusion parameters may be most useful as adjunct tests when routine imaging and early follow-up yield equivocal risk signals, helping refine whether closer surveillance or an earlier discussion on intervention is warranted. However, applying this general information to VSs requires careful attention to how growth is defined and how MRI measurements are acquired and quantified across centers.

Heterogeneity in growth definitions has direct implications for how reported performance metrics should be interpreted. Linear and volumetric criteria capture different aspects of change: linear measurements are broadly feasible but may be less sensitive to small early growth and more affected by measurement variability, whereas volumetry is more sensitive but depends on segmentation methods and software [27,28]. Meanwhile, relative volumetric thresholds can correspond to markedly different absolute growth burdens depending on the baseline tumor size, meaning that the same growth percentage may reflect very different clinical trajectories in small versus larger VSs. As a result, the apparent predictive performance of MRI biomarkers is sensitive to the growth definition used, and restricting analyses to a single definition type would be expected to yield different estimates. In addition, variability in the MRI acquisition may further influence quantitative measurements and limit reproducibility across centers. Consequently, translation will require standardized, clinically meaningful growth definitions and harmonized MRI acquisition and analysis protocols, which require further investigation.

At present, most texture- and perfusion-based biomarkers require dedicated post-processing software and semi-automated segmentation pipelines that are not yet embedded in routine radiology workflows [29]. Clinical deployment would therefore require both the technical training of personnel and regulatory approval, which in turn depends on prospective multicenter validation. In addition, clinically actionable decision thresholds and the cost-effectiveness of biomarker-guided surveillance have not yet been established [30]. Within this framework, integrating MRI biomarkers into clinical workflows may improve the management of VSs by enabling more individualized, risk-stratified surveillance. In practice, implementation may need to prioritize markers derived from routine MRI sequences. First, biomarkers obtainable from standard sequences can be incorporated into radiology reports as structured descriptors. Then, imaging features can be combined with readily available clinical variables to stratify patients into risk groups and guide follow-up intervals. Finally, perfusion MRI may be used selectively as add-on assessments.

Moreover, emerging machine learning models, such as the approach proposed by Hentschel et al. [31], have been used to integrate multimodal inputs and generate probabilistic risk estimates. Chen et al. [32] also proposed a deep learning method; they highlight an emerging shift from static baseline feature extraction to dynamic temporal prediction, which may complement conventional biomarker-based approaches by learning temporal dynamics from serial imaging to predict the future tumor shape. Last but not least, MRI-based biomarkers are non-invasive surrogate markers and cannot replace histopathological assessments when tissue is obtained. Histopathology remains mandatory whenever a surgical resection is performed, and it can help link MRI parameters with tumor microenvironment features (fibrosis and macro-phage infiltration) [33]. Such studies may clarify potential links between imaging findings and pathophysiology and support future validation efforts.

### Limitations

The overall certainty of the evidence is limited by the substantial heterogeneity across studies in growth definitions and MRI protocols. Thus, comparisons are complicated, and conducting a meta-analysis would not be appropriate. Also, five of the ten included studies were retrospective, introducing biases such as incomplete follow-up and non-standardized imaging intervals. Prospective studies such as Sammy et al. [15] remain rare but are essential. Moreover, all included studies were single-center cohorts, which further limits generalizability. Furthermore, the small sample sizes in several studies, combined with the high dimensionality of radiomics features, create a risk of overfitting. This risk is compounded by methodological issues inherent to radiomics, as texture features are sensitive to variations in MRI acquisition and reconstruction, and studies generally lacked standardized feature definitions such as IBSI (Image Biomarker Standardization Initiative) guidelines, which may lead to reproducibility issues. Although some studies reported internal validation, no study explicitly reported independent external validation, exacerbating these problems. Finally, since all included studies reported at least one statistically significant imaging predictor, publication bias cannot be excluded, and the apparent performance may be inflated. These limitations are also reflected in the GRADE assessment, which rated the certainty of evidence as low to very low across biomarker domains, indicating that the confidence in the reported performance estimates is limited and that the findings should be interpreted cautiously.

## 5. Conclusions

Advanced MRI biomarkers, including the texture features, MRI signal intensity and perfusion metrics, show promising but exploratory associations with VS growth, with a discriminatory performance reported across multiple small cohorts, whereas the ADC appears to be more informative for post-treatment response assessments. However, the current evidence is limited by heterogeneous growth definitions, variable MRI protocols, small single-center cohorts, and the absence of external validation. Therefore, these biomarkers are not yet suitable for routine clinical implementation and should primarily guide future prospective, multicenter validation studies with standardized imaging and growth definitions.

## Figures and Tables

**Table 1:** General characteristics included in the study.

Study Number	Author, Year	Patients Number	Growth Number	Age (y)	Mean (Median) FU/MRI Interval * (Mo)	Growth Measurement		MRI Field Strength
						Volume	Linear	
T. Itoyama [12]	2022	64	31	57.9	/	/	>2 mm/year	/
P. Langenhuizen [13]	2020	85	43	/	74	>10%	/	/
Nicholas A George-Jones [14]	2020	53	36	/	6.5 (IQR 5.9–7.4) *	>20%	/	/
Sammy M. Schouten [15]	2024	110	70	58 (48–67)	25 (IQR 17–35)	Subject-specific calculator		3 T
P. Langenhuizen [16]	2020	99	38 (TTE)	58 (IQR 47–66)	/	/	/	/
H. Yamada [17]	2021	31	15	62.9 ± 11.93	27.10 ± 17.27 *	>100 mm^3^/year	/	1.5 T and 3 T
M. C. Kleijwegt [18]	2016	10	9	62 (45–74)	7.6 *	>20%/year	≥2 mm/year	3 T
C. C. Chuang [19]	2012	31	3	/	36.5 (IQR 18–60)	/	/	1.5 T
Herwin Speckter [20]	2019	23	4	51.6 (8.7–75.3)	42.7 (IQR 23.7–80.3)	/	/	3 T
Daniel Lewis [21]	2019	19	7	57.7 (25.7–80.7)	/	/	/	1.5 T

“*” means imaging interval.

**Table 2:** Key technology and metrics reported in the included studies. (**A**) Key metrics reported for texture features; (**B**) key metrics reported for MRI signal intensity; (**C**) key metrics reported for perfusion MRI; and (**D**) key metrics reported for ADC.

**(A)**				
**Author**	**Year**	**Technology**	**Prediction**	**Key Metrics**
T. Itoyama [12]	2022	Idmn	Tumor growth	Mixed model of texture and clinical factors: AUC = 0.69 Idmn: *p* = 0.003
Herwin Speckter [20]	2019	Kurtosis	Tumor growth	71% sensitivity, 78% specificity
Nicholas A George-Jones [14]	2020	First-order, GLCMs	Tumor growth	V > 1.006 cm^3^: 87% sensitivity, 73% specificity AUC = 0.76 V < 1.006 cm^3^: 95% sensitivity, 50% specificity AUC = 0.65
Patrick P. J. H. Langenhuize [13]	2020	GLCMs	Tumor growth	V < 5 cm^3^: 71% sensitivity, 83% specificity AUC = 0.85 V > 5 cm^3^: 83% sensitivity, 82% specificity AUC = 0.99
Patrick P. J. H. Langenhuizen [16]	2020	GLCMs	Tumor TTE	V < 6 cm^3^: 82% sensitivity, 69% specificity V > 6 cm^3^: 77% sensitivity, 89% specificity
**(B)**				
**Author**	**Year**	**Technology**	**Prediction**	**Key Metrics**
H. Yamada [17]	2021	Intensity ratio	Tumor growth	T/Np: 93.33% sensitivity, 75.00% specificity T/Nm: 100.00% sensitivity, 93.75% specificity
T. Itoyama [12]	2022	Minimum signal intensity (T1)	Tumor growth	*p* = 0.016
Herwin Speckter [20]	2019	Minimum signal intensity (T2)	Tumor growth	Correlation coefficient ≈ −0.63
**(C)**				
**Study Number**	**Author,** **Year**	**Technology**	**Prediction**	**Key Metrics**
M. C. Kleijwegt [18]	2016	DSC, ASL	Tumor growth	Hyperintense perfusion characteristics
Daniel Lewis [21]	2019	DCE	Tumor growth	ANOVA, *p* = 0.004
Sammy M. Schouten [15]	2024	DCE	Tumor growth	89% sensitivity, 73% specificity AUC = 0.85
**(D)**				
**Study Number**	**Author,** **Year**	**Technology**	**Prediction**	**Key Metrics**
Sammy M. Schouten [15]	2024	ADC	Tumor growth	Associated with tumor shrinkage (*p* = 0.04) Not predictive of natural tumor growth(*p* = 0.14)
C. C. Chuang [19]	2012	ADC	Tumor growth	Increase in solid tumors Reduction in cystic tumors

V: tumor volume. (A) shows the texture feature-based MRI biomarkers for predicting VS growth or transient tumor enlargement; (B) shows the signal intensity-based biomarkers for predicting VS growth; (C) shows the perfusion MRI biomarkers derived from ASL/DSC/DCE imaging associated with VS growth; and (D) shows ADC-related findings for VS growth prediction.

## Data Availability

No new data were created. Data sharing is not applicable to this article.

## Supplementary Materials

The following supporting information can be downloaded at https://www.mdpi.com/article/10.3390/cancers18020289/s1, Table S1: The NOS scores of ten studies; Table S2: ROBIS assessment of risk of bias in the review process; Table S3: GRADE evidence profile for MRI biomarker domains in VS. References [12,13,14,15,16,17,18,19,20,21] are cited in the Supplementary Materials.

## Abbreviations

The following abbreviations are used in this manuscript:

VS	Vestibular Schwannoma
MRI	Magnetic Resonance Imaging
PICOS	Population, Issue of Interest, Comparison, Outcome, and Study Design
TTE	Transient Tumor Enlargement
NOS	Newcastle–Ottawa Scale
GRADE	Grading of Recommendations, Assessment, Development and Evaluation
ROBIS	Risk of Bias in Systematic Reviews
ADC	Apparent Diffusion Coefficient
GLCM	Gray-Level Co-Occurrence Matrix
Idmn	Inverse Difference Moment Normalized
T/N	Tumor-to-Normal Tissue
T/Nm	Tumor-to-Temporalis Muscle
AUC	Area Under the ROC Curve
ASL	Arterial Spin Labeling
DSC	Dynamic Susceptibility Contrast
DCE	Dynamic Contrast-Enhanced
ANOVA	Analysis of Variance
IBSI	Image Biomarker Standardization Initiative
