# Supplementary material for: MRI-Based Prediction of Vestibular Schwannoma: Systematic Review
Source: Cancers (Basel). 2026 Jan 17;18(2):289. doi: 10.3390/cancers18020289 (PMC12838792; doi:10.3390/cancers18020289)
Supplement: Supplementary file 1 [file cancers-18-00289-s001.zip › Table S1_NOS.pdf]

Reference	Selection				comparabi	Outcome			Score
	Representativeness of the ex-posed cohort	Selection of the nonexposed cohort	Ascertainment of intervention	Demonstration that outcome of interest was not	.Comparability of cohorts on the basis of the	Assessment of outcomes	Was follow-up long enough for outcomes to	Adequacy of follow up to cohorts	
H. Yamada,［17］2021	★	★	★	☆	★☆	★	★	☆	6
T. Itoyama,[12] 2022	★	★	★	☆	★☆	★	★	★	6
Sammy M. Schouten,[15]	★	★	★	☆	★★	★	★	☆	8
M. C. Kleijwegt [18], 2016	★	★	★	☆	★☆	★	★	☆	6
C. C. Chuang,［19］	★	★	★	☆	★★	★	★	★	7
Herwin Speckter,[20]	★	★	★	☆	★★	★	★	☆	7
Nicholas A George-	★	★	★	☆	★★	★	☆	☆	6
Patrick P. J. H. Langenhuizen,[13	★	★	★	☆	★☆	★	★	★	6
Patrick P. J. H. Langenhuizen[	★	★	★	☆	★★	★	★	★	7
Daniel Lewis[21], 2019	★	★	★	☆	★★	★	★	★	8

Legend

Table S1: The NOS scores of ten studies.

This table presents the NOS scale scores of 10 studies, which assess the risk of bias by assessing the quality of cohort studies in three aspects: selectivity, comparability, and outcome.

The **black stars** represent the scores obtained, while the **white stars** represent the scores not obtained. The total score is 9 points, with a score greater than 7 indicating high-quality research
