# Supplementary material for: MRI-Based Prediction of Vestibular Schwannoma: Systematic Review
Source: Cancers (Basel). 2026 Jan 17;18(2):289. doi: 10.3390/cancers18020289 (PMC12838792; doi:10.3390/cancers18020289)
Supplement: Supplementary file 1 [file cancers-18-00289-s001.zip › Table S2_ROBIS.pdf]

Table S2. ROBIS assessment of risk of bias in the review process.

ROBIS Domain	Judgment	Rationale
Study eligibility criteria	Low concern	Eligibility criteria were prespecified (PICOS) and the protocol was prospectively registered (PROSPERO), reducing the risk of post-hoc changes to inclusion/exclusion.
Identification and selection of studies	Some concerns	Core databases were searched using a reproducible strategy and screening was conducted by two reviewers with arbitration. Additional screening targeted unpublished evidence via preprint servers, conference abstract archives, and trial registries. No clearly eligible unpublished negative studies were identified; authors were not contacted for unpublished analyses.
Data collection and study appraisal	Low concern	Data extraction and quality appraisal were conducted in a structured manner, and study-level quality was assessed using the Newcastle–Ottawa Scale, key study characteristics and outcomes were transparently summarized.
Synthesis and findings	Some concerns	Qualitative synthesis was appropriate because heterogeneity precluded pooling: growth definitions varied, MRI field strength differed, and acquisition/post-processing and follow-up context varied. These factors limit cross-study comparability and generalizability of performance estimates.
Overall ROBIS judgment	Some concerns	Main concerns relate to heterogeneity and the inability to formally quantify publication bias in the absence of meta-analysis, despite additional screening of grey literature sources.

ROBIS = Risk Of Bias In Systematic reviews.

Judgments are reported as Low concern / Some concerns / High concern.
