# Supplementary material for: MRI-Based Prediction of Vestibular Schwannoma: Systematic Review
Source: Cancers (Basel). 2026 Jan 17;18(2):289. doi: 10.3390/cancers18020289 (PMC12838792; doi:10.3390/cancers18020289)
Supplement: Supplementary file 1 [file cancers-18-00289-s001.zip › Table S3_GRADE.pdf]

Table S3. GRADE evidence profile for MRI biomarker domains in VS.

Biomarker domain	No. of studies (patients)	Typical design / setting	Summary of findings (as reported)	Risk of bias	Inconsistency	Indirectness	Imprecision	Publication bias	Overall certainty	Main downgrading reasons
Texture features	5 (324)	Mostly retrospective, single-center	AUC ranged from 0.65–0.99 with variable sensitivity/specificity; performance higher in large volume tumor	Very serious	Not Serious	Serious	Not serious	Suspected	Very low	Mostly retrospective, single-center; Overfitting risk; heterogeneous growth definitions/protocols; Small cohorts
Signal-intensity metrics and ratios	3 (118)	Mostly retrospective, single-center	Intensity ratios reported high sensitivity/specificity, other SI metrics reported correlations	Serious	Not Serious	Serious	Not serious	Suspected	Very Low	Mostly retrospective, single-center; Heterogeneous growth definitions/protocols; Small cohorts
Perfusion MRI parameters	3 (139)	prospective, single-center	Reported discrimination up to AUC ~0.85 and significant associations (e.g., Ktrans differences); protocol/post-processing variability	Not Serious	Not Serious	Serious	Not serious	Suspected	Low	Heterogeneous growth definitions/protocols; Small cohorts
ADC	2 (141)	Prospective, single-center	Reported no predictive value for growth but associated with treatment response	Not Serious	Not Serious	Serious	Not serious	Suspected	Low	Heterogeneous growth definitions/protocols; Small cohorts

“serious” and “very serious” indicate downgrading by one or two levels.
